# Resonant pitch and roll silicon gyroscopes with sub-micron-gap slanted electrodes: Breaking the barrier toward high-performance monolithic inertial measurement units

**DOI:** 10.1038/micronano.2016.92

**Published:** 2017-04-24

**Authors:** Haoran Wen, Anosh Daruwalla, Farrokh Ayazi

**Affiliations:** 1 School of Physics, Georgia Institute of Technology, 837 State Street, Atlanta, GA 30332, USA; 2 Department of Electrical and Computer Engineering, Georgia Institute of Technology, 777 Atlantic Drive NW, Atlanta, GA 30332, USA

**Keywords:** anisotropic wet etching, MEMS resonant pitch/roll gyroscope, quadrature cancellation, slanted electrode

## Abstract

This paper presents the design, fabrication, and characterization of a novel high quality factor (*Q*) resonant pitch/roll gyroscope implemented in a 40 μm (100) silicon-on-insulator (SOI) substrate without using the deep reactive-ion etching (DRIE) process. The featured silicon gyroscope has a mode-matched operating frequency of 200 kHz and is the first out-of-plane pitch/roll gyroscope with electrostatic quadrature tuning capability to fully compensate for fabrication non-idealities and variation in SOI thickness. The quadrature tuning is enabled by slanted electrodes with sub-micron capacitive gaps along the (111) plane created by an anisotropic wet etching. The quadrature cancellation enables a 20-fold improvement in the scale factor for a typical fabricated device. Noise measurement of quadrature-cancelled mode-matched devices shows an angle random walk (ARW) of 0.63° √h^−1^ and a bias instability of 37.7° h^−1^, partially limited by the noise of the interface electronics. The elimination of silicon DRIE in the anisotropically wet-etched gyroscope improves the gyroscope robustness against the process variation and reduces the fabrication costs. The use of a slanted electrode for quadrature tuning demonstrates an effective path to reach high-performance in future pitch and roll gyroscope designs for the implementation of single-chip high-precision inertial measurement units (IMUs).

## Introduction

MEMS gyroscopes are self-contained rotation sensors that can be integrated with linear accelerometers to make single-chip inertial measurement units (IMUs)^1–3^. Their small size and low cost have made them successful in various applications ranging from gaming to automotive safety control and have made them promising candidates for personal inertial navigation applications in GPS (global positioning system)-denied environments^1^. Personal navigation requires high-accuracy triaxial rotation sensing on a miniaturized single-package platform. Single-package triaxial gyroscopes can be fabricated through two approaches. In a homogeneous approach, three identical single-axis gyroscope chips are mounted in a single package with different orientations using three-dimensional assembly^4,5^. However, the assembly process usually increases the manufacturing cost and leads to larger form factors as well as alignment inaccuracies. Alternatively, in a single-chip approach, planar gyroscopes are fabricated on a single chip with different designs to achieve both in-plane (IP) and out-of-plane (OOP) sensing structures^6–9^. The single-chip approach enables better manufacturability and a smaller form factor. However, because different designs are involved, it is difficult to obtain consistent high performance for all three sensing axes. Different types of high quality factor (*Q*) resonant yaw gyroscopes (*z* axis) have demonstrated promising performance for navigation applications^10–12^. However, development of high-performance resonant pitch/roll gyroscopes (*x/y* axis) with OOP degrees of freedom is known to be challenging due to fabrication non-idealities.

The performance of a gyroscope is evaluated through its signal-to-noise ratio (SNR). Increasing the SNR of a gyroscope is performed by noise reduction and sensitivity improvement. The best way to achieve large sensitivity in a Coriolis gyroscope is through resonant operation, where the drive and sense modes are matched to have the same exact resonance frequency. When mode-matched, the Coriolis force excites the sense mode at its natural resonance frequency, leading to a *Q*-amplified sense response. However, perfect mode-matching may not be possible due to a frequency mismatch and mode-to-mode coupling (that is, quadrature error) caused by fabrication non-idealities. Quadrature error specifically breaks the eigenvalue degeneracy of the mechanical system, resulting in a veering phenomenon^13^, which appears as a minimum obtainable frequency split between the drive and sense modes. In addition, the quadrature error provides a direct path through which drive-loop noise is carried to the sense mode and becomes a major noise contributor in the sense mode output signal. Considering both effects, quadrature error degrades the SNR of a gyroscope significantly and must be eliminated to achieve high performance. Quadrature cancellation is performed by electrostatic quadrature tuning in resonant yaw gyroscopes^14^. However, in conventional planar pitch and roll gyroscopes fabricated with directional etching techniques, quadrature tuning is typically unavailable. The lack of quadrature tuning capability limits the yield of mode-matchable parts drastically, and even for devices with small as-fabricated quadrature and no obvious veering effect, the residual quadrature still degrades the noise performance significantly^15^. Therefore, quadrature error is considered the biggest obstacle in creating high-performance pitch and roll gyroscopes.

In this paper, we show that slanted electrodes along the (111) anisotropically wet-etched silicon surfaces can be used for efficient quadrature cancellation in pitch/roll gyroscopes with OOP degrees of freedom. In addition, we extend the use of anisotropic wet etching to the fabrication of the entire body of the gyroscope. By doing so, we not only make the frequency split between drive and sense modes insensitive to wafer thickness variations but also eliminate the use of expensive deep reactive-ion etching (DRIE) in device fabrication, reducing both process variation dependency and fabrication cost of the gyroscopes.

## Materials and methods

### Stiffness non-idealities in MEMS gyroscopes

The MEMS Coriolis gyroscope with stiffness non-idealities is represented by a 2-DoF spring-mass-damper system with the equations of motion described as follows:

(1)m1q¨1+b11q˙1+k11q1+k12q2=F1+2λm2Ωq˙2m2q¨2+b22q˙2+k22q2+k21q1=F2−2λm1Ωq˙1

In Equation (1), *m*_1_, *b*_11_, *q*_1_, and *F*_1_ are the effective mass, damping coefficient, displacement, and external actuation force, respectively, associated with mode 1; *m*_2_, *b*_22_, *q*_2_, and *F*_2_ correspond to the same parameters for mode 2. *Ω* is the angular velocity or rate of rotation applied around an axis normal to *q*_1_ and *q*_2_. The factor *λ* is the Coriolis coupling coefficient of the gyroscope, which is determined by the device geometry and mode shape. The *k_ij_* terms are elements of the effective mechanical stiffness matrix of the system. Specifically, *k*_11_ and *k*_22_ are the effective stiffness of mode 1 and mode 2, and *k*_12_=*k*_21_ are the cross-coupling stiffness. The eigen-frequencies of Equation (1) are as follows:

(2)ω1,2=12(k11m1+k22m2±(k11m1-k22m2)2+4k122m1m2)

When operated as a rate gyroscope, one of the modes is actuated into oscillation as the drive mode. In the presence of a rotation *Ω*, the other mode (sense mode) will be excited by the Coriolis force with an amplitude proportional to *Ω*. In yaw gyroscopes, two degenerate IP modes are usually used with both *q*_1_ and *q*_2_ within the device plane^16,17^, whereas in planer pitch and roll gyroscopes, an OOP mode is also involved with displacements normal to the device plane.

For an ideal resonant gyroscope, the stiffness matrix ***K***_m_ is a diagonal matrix satisfying *k*_11_/*m*_1_=*k*_22_/*m*_2_ and *k*_12_=*k*_21_=0, which gives identical eigen-frequencies *ω*_1_=*ω*_2_ for mode 1 and mode 2. In practice, material and fabrication imperfections will introduce a difference between *k*_11_/*m*_1_ and *k*_22_/*m*_2_, causing frequency mismatch. In addition, structural imperfections may produce non-zero stiffness-coupling terms, causing undesired mode-to-mode coupling in addition to the Coriolis coupling, which is called the quadrature error. Based on Equation (2), both frequency mismatch and quadrature error contribute to the total frequency split of the two operational modes. Therefore, maximizing the scale factor by mode-matching requires elimination of both sources of error to bring the frequency split to zero.

In addition to causing mode-split, quadrature errors also affect the sense output directly by creating an undesired path through which the drive mode motion is coupled to the sense mode, causing an inaccurate sense signal and carrying noise from the drive-loop to the sense output. Theoretically, the phase of the quadrature signal is 90° with respect to the Coriolis signal, and its contribution can be eliminated in electronics domain with proper demodulation of the sense output signal with respect to the drive-loop output signal^18^. However, in practice, the quadrature is still considered detrimental to the gyroscope noise performance for several reasons. First, the sense-to-drive phase relationship may vary due to changes in environmental variables such as temperature, especially for non-degenerate modes with different temperature coefficients of frequency. Therefore, maintaining perfect quadrature rejection through demodulation is difficult, and residual quadrature error will cause bias drift and inject additional noise to the sense output, which significantly affects the gyroscope accuracy and stability. Second, with typical fabrication imperfections, quadrature signal levels can be as high as Coriolis signals due to rotations above thousands of degrees per second. Consequently, the gain of the front-end amplifiers must be kept small to avoid saturation before demodulation, resulting in a more pronounced input-referred noise contribution from the demodulation circuit. Especially for designs utilizing large actuations^19^, the quadrature level increases with drive amplitude and significantly limits the sense signal amplification, leading to a low SNR dominated by the electrical noise from the interface circuit.

### Quadrature tuning in pitch/roll gyroscopes

Electrostatic tuning through capacitive transduction can provide compensations for stiffness non-idealities on the device level. In capacitive transduction, fixed electrodes are placed near the resonant structure of the gyroscope, forming parallel-plate capacitors. Electrical energy stored in the capacitor between the resonator and a fixed electrode is a function of *q*_1_ and/or *q*_2_, which gives rise to the following electrostatic stiffness matrix:

Ke=[ke11ke12ke21ke22]=−[∂2Ue∂q12∂2Ue∂q1∂q2∂2Ue∂q1∂q2∂2Ue∂q22](3)Ke=−V22[∂2C(q1,q2)∂q12∂2C(q1,q2)∂q1∂q2∂2C(q1,q2)∂q1∂q2∂2C(q1,q2)∂q22]

where *V* is the DC voltage difference between the resonator and the fixed electrode, typically equal to the difference between the device polarization voltage *V*_P_ and the DC voltage applied to the electrode. The overall electromechanical stiffness of a gyroscope is the superposition of ***K***_m_ and ***K***_e_ from all electrodes, as follows:

(4)Ktotal=Km+∑Ke

**Figure 1:**
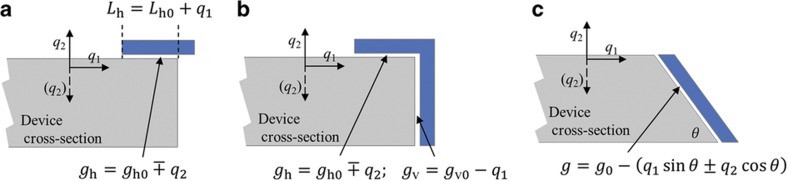
Cross-sectional view of different types of electrodes in out-of-plane gyroscopes. (**a**) Horizontal electrode parallel to the top surface of the device. (**b**) Right-angle electrode formed by combined horizontal and vertical electrodes. (**c**) Slanted electrode.

By changing tuning voltages at different electrodes, the overall stiffness can be adjusted. However, the effectiveness of electrostatic tuning in pitch and roll gyroscopes with both IP and OOP modes is somewhat limited. In conventional gyroscopes fabricated with a directional etching process, electrodes are placed either horizontally or vertically. Assuming *q*_1_ is IP and *q*_2_ is OOP, then the horizontal electrode capacitance (Figure 1a), for example, is given by the following equation:

(5)Ch=ε0Ah(q1)gh(q2)=ε0Wh0(Lh0+q1)gh0∓q2

where *W*_h0_, *L*_h0_, and *g*_h0_ are the width, length of the overlapping area, and gap size when the gyroscope is at rest. For small displacements, the second derivatives corresponding to electrostatic stiffness terms are as follows:

(6)∂2Ch∂q12=0,∂2Ch∂q1∂q2≈Ch0gh01Lh0,∂2Ch∂q22≈Ch0gh01gh0

where *C*_h0_=(*εW*_h0_*L*_h0_)*/*g_h0_ is the rest capacitance of the electrode. To achieve efficient transduction, large capacitance is necessary. Therefore, a large electrode area (*W* and *L*) and small gap size (*g*) are favorable. In typical MEMS gyroscopes, an *L/g* ratio ~100 is necessary to obtain sufficient tuning with reasonable voltages^12,17^. Comparing each of the terms in Equation (6), the horizontal electrode provides efficient OOP frequency tuning but no IP frequency tuning and a negligible cross-coupling tuning. In other words, for tuning purposes, the horizontal electrode capacitance is mainly a function of *q*_2_, and the contribution from the change of area under *q*_1_ is negligible. Similarly, a vertical electrode capacitance is mainly a function of *q*_1_, and tuning effects associated with *q*_2_ are negligible. Consequently, any combination of horizontal and vertical electrodes will produce a capacitance as a separable function of *q*_1_ and *q*_2_, namely, *C*(*q*_1_,*q*_2_)≈*C*_1_(*q*_1_)+*C*_2_(*q*_2_). For example, in a right-angle electrode (Figure 1b), the electrical energy (neglecting the small fringing field) is given by the following equation:

(7)Ue≈V22(Cvertical+Chorizontal)=ε0V22(Ahgh+Avgv)

Assuming without the loss of generality that both horizontal and vertical capacitors have the same rest gap size *g*_0_, a series expansion results in the following equations:

Ue=ε0V22(Ahg0−q1+Avg0∓q2)(8)Ue≈ε0V22g0[Ah+Av+Ahq1g0±Avq2g0+Ah(q1g0)2+Av(q2g0)2+⋯]

From Equation (3), the corresponding **K**_e_ is diagonal:

(9)Ke=[ke11ke12ke21ke22]=−ε0V2g03[Ah00Av]

which means that an IP or OOP frequency mismatch may be compensated, but no quadrature tuning is achievable.

In contrast, a slanted surface electrode introduces a well-defined relationship between IP and OOP degrees of freedom. Capacitive electrodes along the slanted surfaces have capacitive gaps that are affected by both IP and OOP motions (Figure 1c). When placed at the antinodal location of both modes, the energy stored in an electrode with slanting angle *θ* is:

Ue=ε0AV22gs=ε0AV22[g0−(q1sinθ±q2cosθ)](10)Ue≈ε0AV22g0[1+q1sinθ±q2cosθg0+(q1sinθ±q2cosθg0)2+⋯]

Correspondingly, the electrostatic stiffness matrix ***K***_e_ is:

(11)Ke=[ke11ke12ke21ke22]=−ε0AV2g03[sin2θ±sinθcosθ±sinθcosθcos2θ]

As shown in Equation (11), the electrostatic stiffness matrix has non-zero off-diagonal terms that are suitable for quadrature cancellation for a non-zero *θ*. When *θ*=45°, optimized quadrature tuning is obtained where the off-diagonal terms are maximized, and the diagonal terms are identical. When *θ* is not 45°, quadrature tuning must be accompanied by proper frequency mismatch tuning to compensate for the effect of non-identical diagonal terms.

### Gyroscope design and simulation

A practical way to achieve a slanted surface in microfabrication is through anisotropic wet etching of (100) single crystal silicon (SCS). Anisotropic wet etching of SCS is self-bounded by the (111) crystal planes. For (100) SCS wafers, the (111) planes are slanted with a well-defined angle of 54.74° from the horizontal plane. By Equation (11), the corresponding electrostatic stiffness matrix is:

(12)Ke=[ke11ke12ke21ke22]=−ε0AV2g03[0.67±0.47±0.470.33]

which shows nearly optimal off-diagonal terms for quadrature tuning.

In addition to providing slanted surfaces, the self-bounded nature of anisotropic wet etching also allows precise control of the surface finish and final geometry, which makes it suitable for the batch fabrication of geometrically sensitive MEMS devices, such as MEMS gyroscopes, and for replacing more expensive fabrication processes such as DRIE. Considering the advantages of anisotropic wet etching, a novel SCS pitch/roll gyroscope design with slanted quadrature electrodes has been developed, in which the entire resonator is formed by the anisotropic wet etching of SCS.

**Figure 2:**
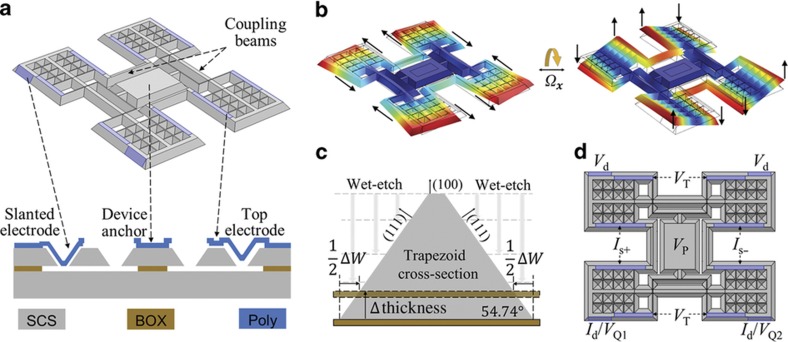
Anisotropically wet-etched gyroscope geometry and mode shapes. (**a**) Device geometry: colored regions indicate the transduction surfaces. Cross-sectional view shows the shape of the gyroscope body and polysilicon electrode at different locations. (**b**) Mode shapes of the in-plane mode and the out-of-plane mode. (**c**) Cross-section of a coupling beam showing the linear dependency of the beam bottom width on the device thickness. In the presence of a device thickness variation, the distance from the top surface to the actual BOX layer (dotted outline) differs from the distance to the ideal BOX layer (solid outline), causing the bottom width of the beam to vary correspondingly. (**d**) Electrode configuration of the gyroscope. Differential sense output is enabled by using the out-of-plane (OOP) mode as the sense mode to reject common-mode errors.

The gyroscope design features four proof-masses coupled through two pairs of coupling beams. Figure 2a shows the resonant structure of a typical design with a 40 μm thickness. The two resonance modes of the gyroscope are shown in Figure 2b. In one mode, the beams bend in the IP directions, causing each mass to rotate IP around connecting points to one pair of the beams. In the other mode, one pair of beams bends in the OOP direction, and the other pair of beams deforms torsionally, causing each mass to rotate OOP around axes coinciding with the torsional beams. These two modes are Coriolis-coupled, as shown in Figure 2b. One of the modes can serve as the drive mode of the gyroscope, and the other mode can serve as the sense mode of the gyroscope. Dimensions of the proof mass and coupling beams are designed to satisfy the frequency-matching constraints of the IP and OOP modes at a high resonance frequency of 200 kHz.

Fabricating the entire resonator with anisotropic wet etching not only provides slanted surfaces for placing quadrature electrodes but also improves the robustness of the design against the thickness variation of the starting silicon-on-insulator (SOI) substrate. Device thickness variation is one of the common non-idealities in pitch and roll gyroscopes fabricated on SOI wafers. A variation of ±0.5 μm is common for commercial SOI wafers with tens-of-micron-thick device layers, and in the case of heavily doped wafers, this variation can even be a few microns. In conventional designs, the frequency of the IP mode is normally independent of thickness, and the frequency of the OOP mode has a linear dependence on the device layer thickness. The variation in thickness will introduce a large frequency mismatch between the two modes, especially for higher frequency designs, where electrostatic compensation becomes insufficient and the operational modes become unmatchable.

The anisotropically wet-etched gyroscope features an isosceles trapezoid cross-section for the coupling beams (Figure 2c). The top width of the trapezoid is defined by lithography, whereas the bottom width is determined by both the top width and the thickness of the device, thus introducing a thickness dependence to the IP resonance frequency of the gyroscope. Therefore, the IP and OOP modes track each other over device thickness variations, making the frequency mismatch less sensitive to the thickness variability of the SOI wafer. Consequently, we can go to thicker designs with higher operating frequencies and better environmental robustness without requiring extensive frequency mismatch compensation.

Polysilicon electrodes with narrow gaps are placed along the SCS device surfaces for capacitive actuation, readout, and tuning. Figure 2d shows the electrode configuration of the gyroscope. In the specific design, the IP mode is chosen to be the drive mode, and the OOP mode is chosen to be the sense mode with a differential sense output. A pair of slanted electrodes is used to actuate the IP drive mode while excitation of the OOP sense mode is avoided due to opposite mode symmetries at the two electrodes. Another two slanted electrodes are used for either the drive mode current output or the electrostatic quadrature tuning. As labeled in Figure 2d, *V*_Q1_ and *V*_Q2_ have opposite quadrature tuning effects. Depending on the type of quadrature (positive or negative) in a specific device, one of the two slanted electrodes is used for quadrature cancellation with a quadrature tuning voltage *V*_Q_, and the other electrode is used for the output of the drive mode with a zero DC voltage. Horizontal electrodes on top of the device are used for differential sense output and sense mode frequency tuning. Capacitive gaps (~500 nm) are used to achieve sufficient tuning with *V*_P_ up to 20 V. Thickness of the polysilicon electrode is chosen to be 7.5 μm, which gives a pull-in voltage of 25 V, allowing more than a 25% margin from *V*_P_. The sense mode is designed to be 2 kHz lower than the drive mode when the device is biased at *V*_P_, and all other electrodes are at 0 V to ensure mode-matching capability through OOP frequency tuning in the presence of process variations.

**Figure 3:**
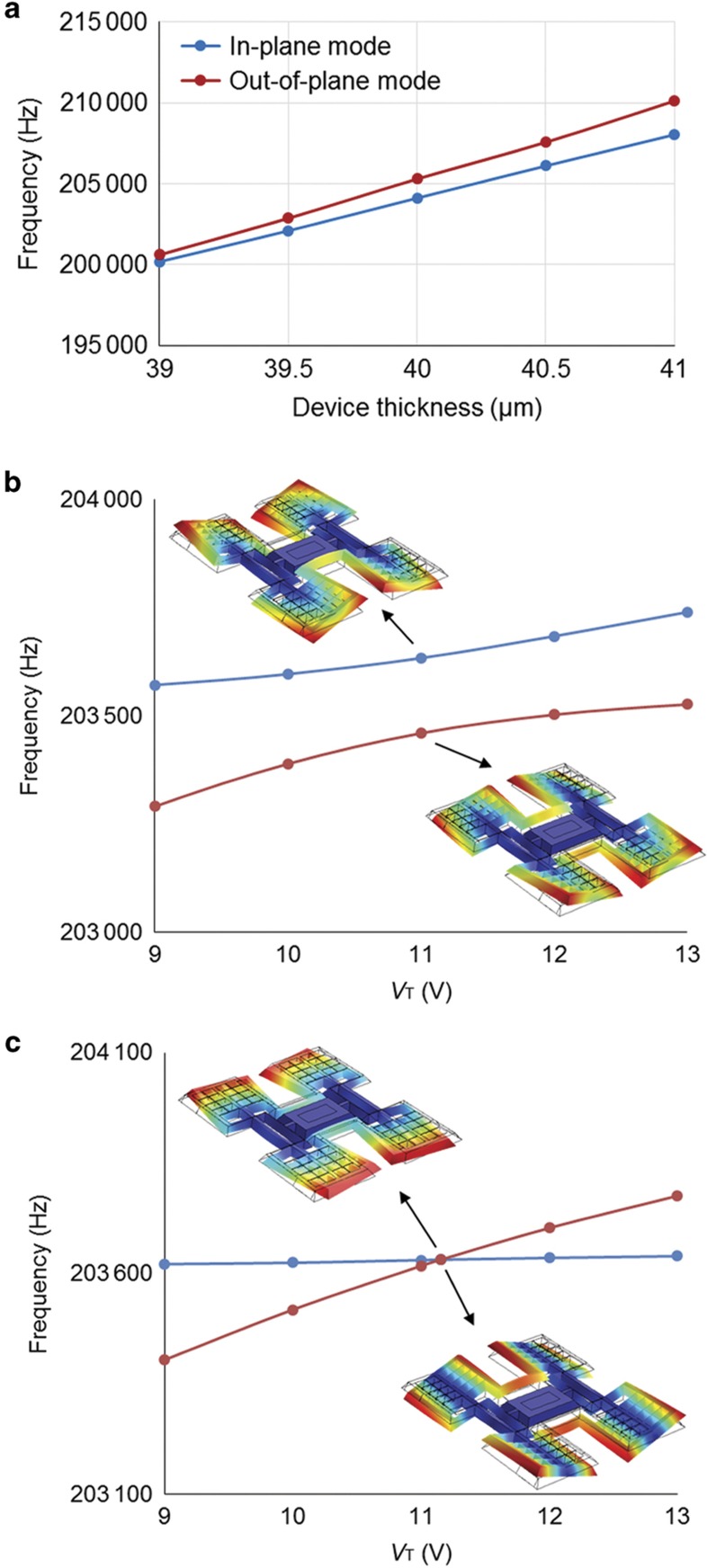
Finite element method simulation results. (**a**) Frequency variation with different device thickness. (**b** and **c**) Frequency tuning without and with electrostatic quadrature cancellation.

To verify the effectiveness of electrostatic mode-matching and thickness variation insensitivity, finite element method simulations are performed on an imperfect anisotropically wet-etched gyroscope model using COMSOL Multiphysics. Figure 3a shows the thickness dependence of the operational mode frequencies. Across a 2 μm thickness variation, the resonance frequencies change significantly, whereas the frequency difference between the two operational modes remains relatively small.

The mode-matching behavior is shown in Figures 3b and c. Geometric imperfection is added intentionally in the model to simulate non-idealities causing quadrature. In the simulations, the sense mode frequency is tuned up by increasing the tuning voltage *V*_T_ to reduce frequency split. As shown in Figure 3b, in the presence of quadrature, a frequency veering phenomenon is observed, and the two eigen-modes cannot be matched. As the two modes become close, the IP and OOP mode shapes become mixed through mode-to-mode coupling. When the frequency mismatch is fully compensated, a minimum frequency split is found, which is due to quadrature only. At the minimum frequency split, the mode-to-mode coupling is maximized, and the two mode shapes become indistinguishable. As the tuning voltage increases further, the frequency split becomes larger, and the mode shape mixing decreases. In contrast, when a proper quadrature tuning voltage *V*_Q_ is applied to the slanted quadrature electrode, mode-to-mode coupling can be eliminated. As shown in Figure 3c, when the two modes are decoupled with proper *V*_Q_, the OOP mode can be tuned independently, and perfect mode-matching is achievable.

Energy dissipation in the gyroscope is also simulated, showing a quality factor of 24 000 for the IP mode and 30 000 for the OOP mode at 24 °C in the sub-100 mTorr environment, both limited by thermoelastic damping.

### Fabrication

**Figure 4:**
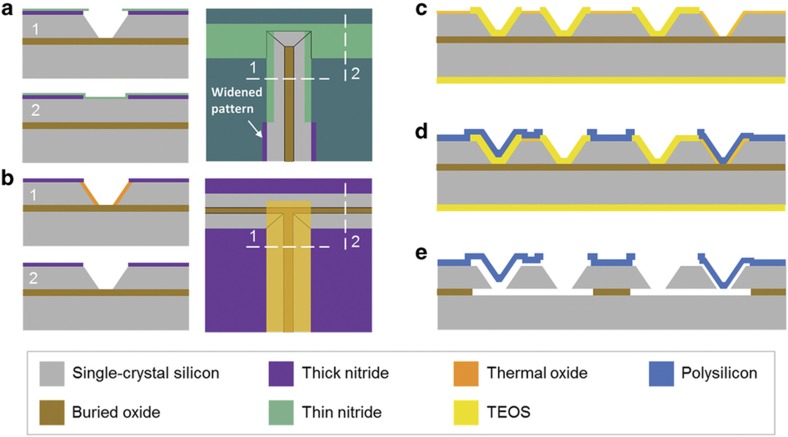
Fabrication process flow for the anisotropically wet-etched pitch/roll silicon gyroscope. (**a**) First wet etching with self-aligned masks. (**b**) Second wet etching after local oxidation. (**c**) TEOS deposition and patterning, and thermal oxidation. (**d**) Thermal oxide patterning, polysilicon deposition and patterning. (**e**) Release. TEOS, tetraethyl orthosilicate.

Figure 4 shows the fabrication process flow for the anisotropically wet-etched resonant gyroscope. The gyroscope is fabricated on a 40 μm (100) SOI wafer using a five-mask process. The entire resonator and electrode anchors are formed during the anisotropic wet etching steps. KOH solution (45%) is chosen as the wet etchant for its high crystalline selectivity. A two-mask LOCOS method^20^ is used to protect the convex corners during the wet etching steps, with modifications made to eliminate misalignment-caused variations in the final geometry. In the self-aligned process, the complete wet etching pattern is defined on a first thick nitride mask layer. Before performing wet etching, a second thin nitride layer is deposited and patterned to partially cover the first mask pattern at the convex corner regions. The patterns on the thin nitride mask are widened at the non-convex-corner regions to expose patterns on the first mask. Wet etching is then performed, which forms trenches aligned to the thick nitride mask (Figure 4a). The finished trench dimensions are independent of the second mask, thus insensitive to misalignment between the two nitride masks. Following Ref. 20, local oxidation is performed to protect the (111) surfaces produced in the first wet-etching step. The thin nitride mask is then removed to expose the remaining pattern on the thick nitride mask. Finally, a second wet-etching step is performed, forming the final SCS structures with intact convex corners (Figure 4b).

After the SCS structures are defined, the wet-etching masks are removed and polysilicon and sacrificial-oxide surface micromachining steps^21^ are used to form the electrodes. First, a thick oxide is deposited conformally using the tetraethyl orthosilicate (TEOS) process and patterned to selectively expose the SCS transduction region both on the top and slanted surfaces. Second, the wafer is thermally oxidized to form a thin sacrificial oxide over the transduction region. The thin oxide defines the transduction gap size, and the thick TEOS forms a large separation between the resonator and the electrodes to avoid undesired transduction (Figure 4c). The thermal oxide is selectively removed on the anchors, and a polysilicon layer is deposited and patterned to form the electrodes (Figure 4d). Finally, the device is released in hydrofluoric acid solution to suspend the resonator (Figure 4e). Released devices are dried using a supercritical dryer to avoid potential stiction issues.

**Figure 5:**
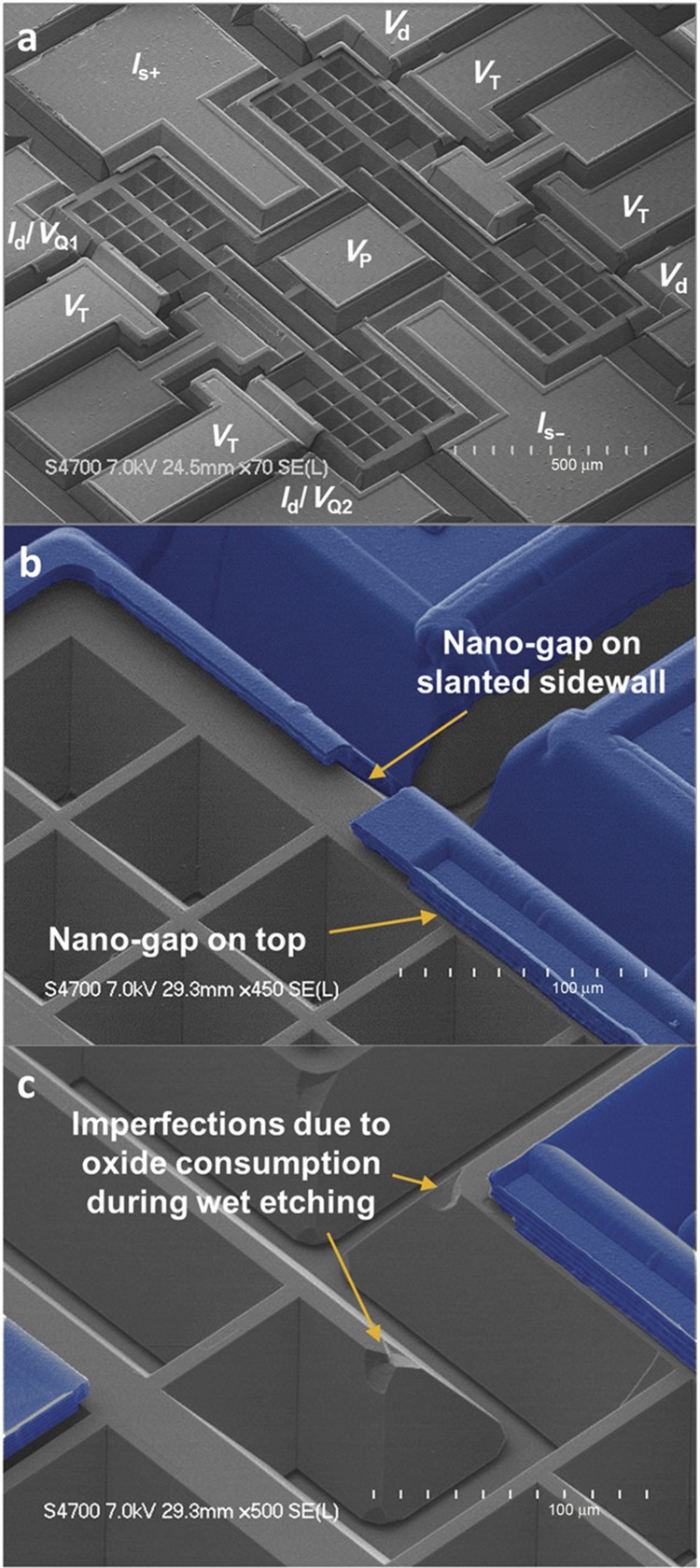
SEM views of a fabricated gyroscope. (**a**) Electrical connections are labeled on the polysilicon pads, showing the electrode configuration used in measurements. (**b** and **c**) Polysilicon electrodes are shown in blue. Capacitive gaps of 540 and 510 nm are formed for the slanted and top electrodes, respectively, via thermal oxidation. SEM, scanning electron microscopy.

Figure 5 shows the SEM (scanning electron microscopy) pictures of a fabricated gyroscope. The self-confined nature of the anisotropic wet etching results in very smooth slanted sidewalls. Sub-micron transduction gaps are selectively formed both on the top and on the slanted sidewalls of the device with the gap size of 510 nm for the top electrodes and 540 nm for the slanted electrodes defined by the thickness of the sacrificial thermal oxide. The thickness of the deposited polysilicon electrode layer is 7.5 μm as designed, which gives it enough stiffness for the designed operating voltages. Convex corners are mostly protected, although small imperfections (tolerable) are found due to the protecting oxide being slightly consumed during excessive KOH etching.

## Results and discussion

### Resonator characterization

**Figure 6:**
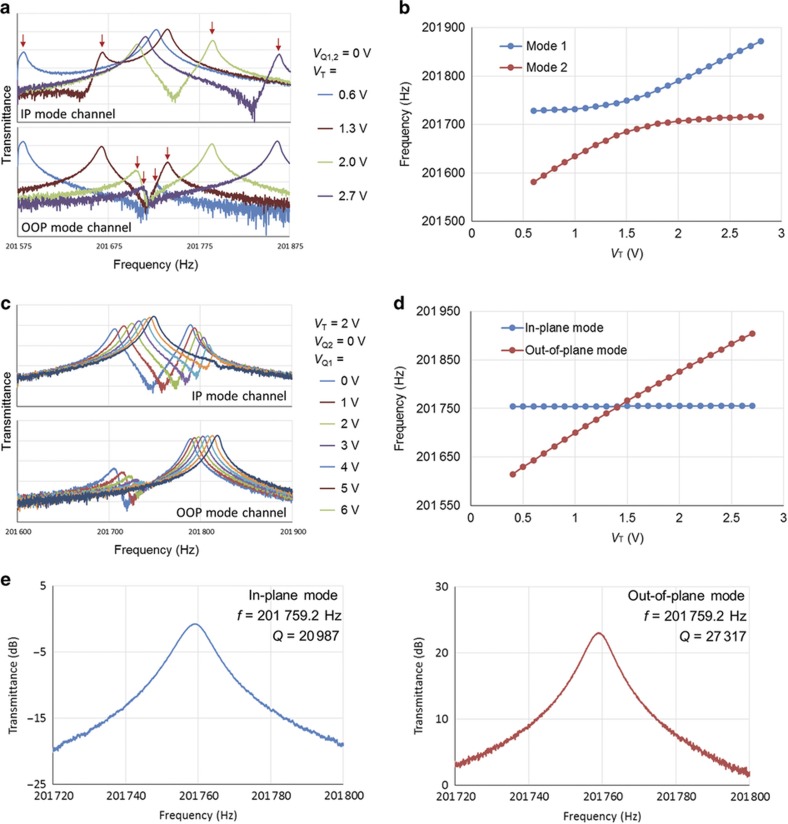
Typical electrostatic tuning behavior of the anisotropically wet-etched out-of-plane gyroscope. (**a**) Frequency response to different values of *V*_T_ without applying quadrature cancellation. Cross-coupling peaks are pointed out with red arrows. (**b**) Resonance frequencies of the two modes versus tuning voltage *V*_T_ without applying *V*_Q_. A minimum frequency split of 64 Hz is observed. (**c**) Frequency response to different quadrature tuning voltage V_Q1_ with fixed *V*_T_=2 V. (**d**) Resonance frequencies of the two modes versus tuning voltage *V*_T_ with optimized *V*_Q_. (**e**) Mode-matched peaks of the in-plane and out-of-plane modes.

The quadrature tuning and mode-matching behaviors of fabricated resonant gyroscopes are characterized in a vacuum chamber using a four-port network analyzer (Agilent 5071C). The IP mode and OOP mode are actuated using separate channels, and the frequency response of each channel is recorded. Figure 6 shows the typical frequency response of a device with different electrostatic tuning conditions. For this specific device, the body of the resonator is biased at *V*_P_=19 V. First, the OOP tuning voltage *V*_T_ is increased to reduce the frequency mismatch while all other electrodes are at 0 V. As shown in Figure 6a, cross-coupling peaks exist in both channels (labeled with red arrows). When the modes are tuned closer in frequency, the quadrature peak becomes more pronounced as the mode shape mixing increases. Figure 6b shows the corresponding resonance frequencies of each mode at different OOP tuning voltages, and a frequency veering phenomenon is observed where the minimum frequency split is found to be 64 Hz.

The effect of quadrature tuning is shown in Figure 6c. *V*_T_ is fixed at 2 V, and the quadrature tuning voltage *V*_Q1_ is adjusted to reduce quadrature while all other electrodes are at 0 V. As the quadrature tuning voltage increases, the cross-coupling peaks in both channels are reduced efficiently, and the quadrature level becomes close to minimized at *V*_Q1_=6 V. The optimized tuning voltages are found to be *V*_T_=1.416 V and *V*_Q1_=6.86 V by iterative fine adjustment of *V*_T_ and *V*_Q1_ to achieve a zero frequency split. Figure 6d shows the OOP frequency tuning behavior with *V*_Q1_=6.86 V. The OOP mode is tuned independently while the IP mode frequency remains constant. No frequency veering phenomenon is observed and mode-matching is achieved, which confirms the elimination of mode-to-mode coupling. Figure 6e shows the mode-matched resonance peaks with less than 0.1 Hz measurement tolerance. Quality factors are measured as 21 000 for the IP mode and 27 000 for the OOP mode, in close agreement with the simulated values.

### Gyroscope performance characterization

The device is interfaced with discrete electronics and an HF2LI lock-in amplifier from Zurich Instruments (Zurich, Switzerland) for performance characterization. Feed-through cancellation circuits are included to minimize the effect of feed-through capacitance between input and output electrodes^22^.

**Figure 7:**
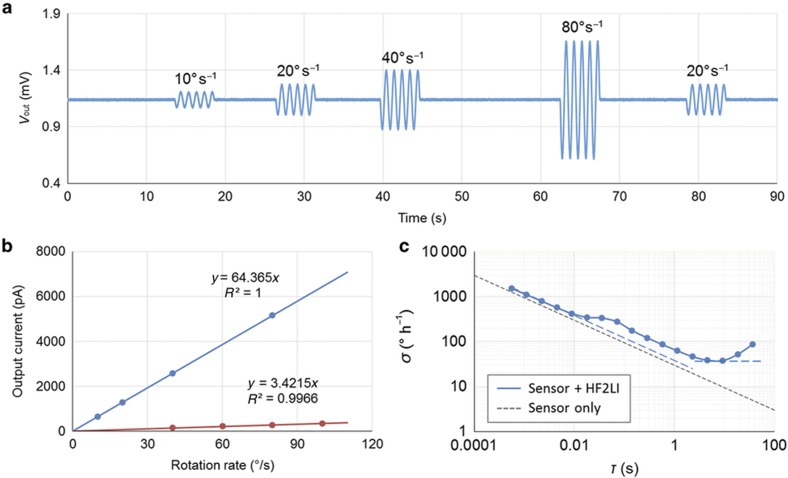
Resonant gyroscope performance characterization. (**a**) Mode-matched gyroscope output response to different rotation rates. The plot shows 90 s of continuous measurement. A consistent scale factor is measured, and no ZRO drift is observed. (**b**) Measured scale factor of the gyroscope with and without quadrature cancellation. Voltage output is converted into current knowing the TIA gain. Mode-matching after quadrature cancellation leads to a 20-fold improvement in the scale factor. (**c**) Allan deviation plot of mode-matched gyroscope. An ARW of 0.63° (√h)^−1^ and a bias instability of 37.7° h^−1^ are measured. ARW, angle random walk; TIA, transimpedence amplifier; ZRO, zero rate output.

The angular rate response of the gyroscope is characterized by applying 10–80° s^−1^ rotation rates (Figure 7a). The mode-matched scale factor of the gyroscope is measured as 64.4 pA (° s^−1^)^−1^. For comparison, sensitivity is also measured with a minimum frequency split without quadrature tuning, which gives a scale factor of 3.42 pA (° s^−1^)^−1^, indicating ~20-fold improvement in sensitivity through quadrature cancellation and mode-matching (Figure 7b). A trade-off between mode-matching and bandwidth exists. By going to higher frequency designs, mode-matched bandwidth can be improved compared to low frequency designs. The 3 dB bandwidth of the resonant gyroscope presented is ~8 Hz. This bandwidth can be further increased by optimizing the design to have higher operating frequencies. Ultimately, closed-looped operations^23^ can be used with the resonant gyroscope for applications requiring very high bandwidth.

Figure 7c shows the measured Allan deviation of the mode-matched resonant gyroscope. The complete gyroscope system exhibits an angle random walk (ARW) of 0.63° √h^−1^ (0.01°s^−1 ^√Hz^−1^) and a bias instability of 37.7° h^−1^. Noise from the electronics is estimated to be 0.38° √h^−1^. Further improvements of the electrical noise would reduce the ARW to its theoretical limit of 0.5° √h^−1^ corresponding to the mechanical thermal noise of the design (black dashed trace in Figure 7c). The irregular bump at ~0.017 s is possibly due to 60 Hz power line interference and feed-through on the evaluation board.

For some emerging applications, such as inertial navigation, an ARW less than 0.1° √h^−1^ and a bias instability less than 1° h^−1^ are desirable. A 5-fold improvement in ARW can be achieved in the anisotropically wet-etched gyroscope to meet the high-end requirement by increasing the effective mass and drive amplitude through device geometry and transduction optimization. The minimum achievable bias instability is determined by flicker noise in the interface circuit. Quadrature cancellation improves the bias instability of the gyroscope by decreasing noise coupling between the drive-loop and the sense output. Optimization of interface circuitry design will further reduce flicker noise in the system and provide a lower bias instability floor. In addition to random noise errors, deterministic errors due to environmental factors such as linear vibrations and temperature variations will cause a drift rate ramp^24^, which may prevent the gyroscope from reaching the actual bias instability floor. With high-stiffness designs having high resonance frequencies, effects of linear vibrations will be suppressed^4^. Temperature effects can be reduced by incorporating automatic mode-matching circuits, which minimizes bias drift by providing in-run dynamic tuning and mode-matching of the gyroscope at all times^25^.

## Conclusions

This paper presents a novel approach to quadrature cancellation in resonant pitch and roll gyroscopes. By introducing a slanted electrode, electrostatic quadrature cancellation is achieved in the planar pitch and roll gyroscopes with OOP degrees of freedom. A new type of resonant gyroscope featuring slanted quadrature tuning electrodes is presented with its entire resonant structure fabricated by anisotropic wet etching of the SCS. Simulation and measurement results show that the anisotropically wet-etched design not only allows quadrature tuning but also presents a small thickness dependence of frequency split. Successful electrostatic quadrature cancellation is demonstrated, for the first time, on fabricated OOP pitch/roll gyroscopes. The elimination of quadrature allows mode-matching of the gyroscope, resulting in ~20-fold improvement in sensitivity. Measurements show an ARW of 0.63° √h^−1^ and a bias instability of 37.7° h^−1^, which are partially limited by noise in the electronics. Table 1 highlights the device specifications and performance metrics of the gyroscope presented. Improvements in noise measurement are expected with a better testing setup. Optimization of the gyroscope and circuity designs can further reduce the ARW and bias instability of the system to achieve higher performance for high-end applications.

The results presented in this paper show, for the first time, an efficient way to achieve quadrature cancellation on the device level for OOP pitch and roll gyroscopes. In the future, the use of slanted electrodes can be applied to various planar pitch and roll gyroscope designs to overcome quadrature errors, making it possible to achieve a high-performance monolithic IMU.

## Figures and Tables

**Table 1:** Performance metrics and specifications of the anisotropically wet-etched resonant pitch/roll gyroscope

Parameter	Value	Unit
Resonator size	1×1.3	mm×mm
Top gap size	510	nm
Slanted gap size	540	nm
Resonance frequency	201.7592	kHz
		
Mode-matched Q		
Drive	21 000	
Sense	27 000	
		
*Δf*_min_		
w/o *V*_Q_	64	Hz
w/ *V*_Q_	<0.1	
		
Scale factor		
w/o *V*_Q_	3.42	pA (° s^−1^)^−1^
w/ *V*_Q_	64.4	
		
ARW	0.63	° √h^−1^
Bias instability	37.7	° h^−1^

Abbreviations: ARW, angle random walk; w/, with; w/o, without.
